# Unraveling the Relevant Length and Time Scales of Elastomers in a High Strain Rate Test

**DOI:** 10.1021/acs.macromol.5c02872

**Published:** 2026-01-22

**Authors:** Katherine M. Evans, Dustin A. Baird, Polette J. Centellas, Christopher L. Soles, Edwin P. Chan

**Affiliations:** Materials Science and Engineering Division, 10833 National Institute of Standards and Technology, 100 Bureau Dr, Gaithersburg, Maryland 20899, United States

## Abstract

This study uses a recently developed cavitation approach, laser-induced membrane expansion (LIME), to study the high strain rate mechanical properties of three elastomers: poly­(styrene-*b*-isoprene-*b*-styrene) (SIS), cross-linked linear polydimethylsiloxane (*l*PDMS), and cross-linked bottlebrush PDMS (*b*PDMS) with different network topologies. The cavitation event was modeled as a damped harmonic oscillator to quantify the shear modulus and dissipation of the elastomer as a function of strain rate. Interestingly, the high strain rate shear moduli obtained from LIME of the three elastomers were similar, whereas they had markedly different shear moduli measured under quasi-static conditions. The length of the elastically active polymer during the cavitation event was also calculated, which is an important parameter for understanding the amount of energy dissipated in this high strain rate test. The difference in fundamental polymer dynamics that results in different mechanical properties as measured at high versus low strain rates has important implications for designing polymer networks that store, release, and dissipate energy effectively during high-rate deformation events.

## Graphical abstract

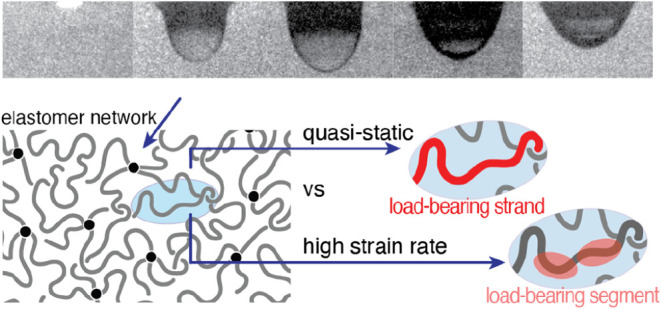

## Introduction

Elastomers are an intriguing class of polymers because they display nonlinear mechanical behavior including hyperelasticity, strain-, strain rate- and temperature-dependence.^1–5^ In particular, the strain-rate-dependent mechanical properties can result in brittle to ductile to rubbery behavior.^6–8^ This presents unique opportunities in terms of deploying these materials but also presents challenges in understanding their behavior. For applications in advanced manufacturing, medical devices, impact protection, and more, an elastomer may need to withstand or have to respond to a range of strain rate conditions without failure.^2,8–13^ Thus, it is essential to study both quasi-static (<1 s^–1^) properties and high-rate (>10 s^–1^) mechanical properties.

In a quasi-static mechanical test, the deformation is slowly applied to a material. The deformation is, therefore, transmitted across the material over a variety of length scales: bonds can stretch, rotate, and rupture, monomers can slide past one another, chain segments can elongate and disentangle, etc. The mechanical properties represent the response of the entire material across the broad range of polymer length scales.^8,14^

In contrast to a quasi-static test, there is limited time for the entire material to respond in a high strain rate test. Rather, only polymer segments of specific lengths can respond within this limited time. The exact length scales, that are defined by polymer-specific properties such as the Kuhn length, monomeric friction coefficient, segmental dynamics, Rouse time, etc., capable of responding will depend on the strain rate, and other experimental conditions.^8,14^ As a result, the mechanical properties of a polymer can be significantly different at high strain rate compared to a quasi-static test since it is expected that different polymer length scales will dominate.

To study the specific length scales that contribute to the mechanical response of a polymer at high strain rates, we apply laser-induced membrane expansion (LIME) to measure the mechanical properties of poly­(styrene-*b*-isoprene-*b*-styrene) (SIS), cross-linked linear polydimethylsiloxane (*l*PDMS), and cross-linked bottlebrush PDMS (*b*PDMS) elastomers. LIME, which is a high strain rate cavitation measurement,^15^ shares similarities to laser-induced cavitation^16^ or inertial microcavitation.^17^ Previously, LIME was used to estimate the shear modulus of a silicone elastomer by measuring the rate of expansion of a bubble and fitting it to an empirically derived model. While this model effectively quantified the high strain rate modulus, it provided little insight into the underlying physics, as it simply analyzed bubble expansion, ignoring the bubble contraction process. Here, we refine the LIME technique by showing that the entire high-rate cavitation event of all three elastomers can be modeled as damped harmonic oscillators. The harmonic oscillator model provides insight into how energy is stored (storage modulus) or dissipated (dissipation factor) due to material and geometric properties. We discuss the relevant polymer length scales that can be accessed in these high strain rate experiments, which are significantly smaller compared to the length scales probed by quasi-static mechanical tests.

## Results

LIME is derived from laser ablation-based techniques such as laser-induced forward transfer (LIFT) and laser-induced projectile impact testing (LIPIT), both of which have been used to study the mechanical properties of polymeric films.^18–23^ A schematic of the LIME experiment is shown in Figure 1a. In a LIME experiment, an ablation target is first created. This target consists of three layers: a glass coverslip, a thin layer of gold (≈30 nm), and the polymer film (10 to 185 μm). A single laser pulse is then focused on the gold to generate a plasma (see Figure 1b, panel (i)). The plasma causes the polymer membrane to expand rapidly, which is captured using an ultrafast camera with a frame rate of ≈10^6^ s^–1^.

**Figure 1:**
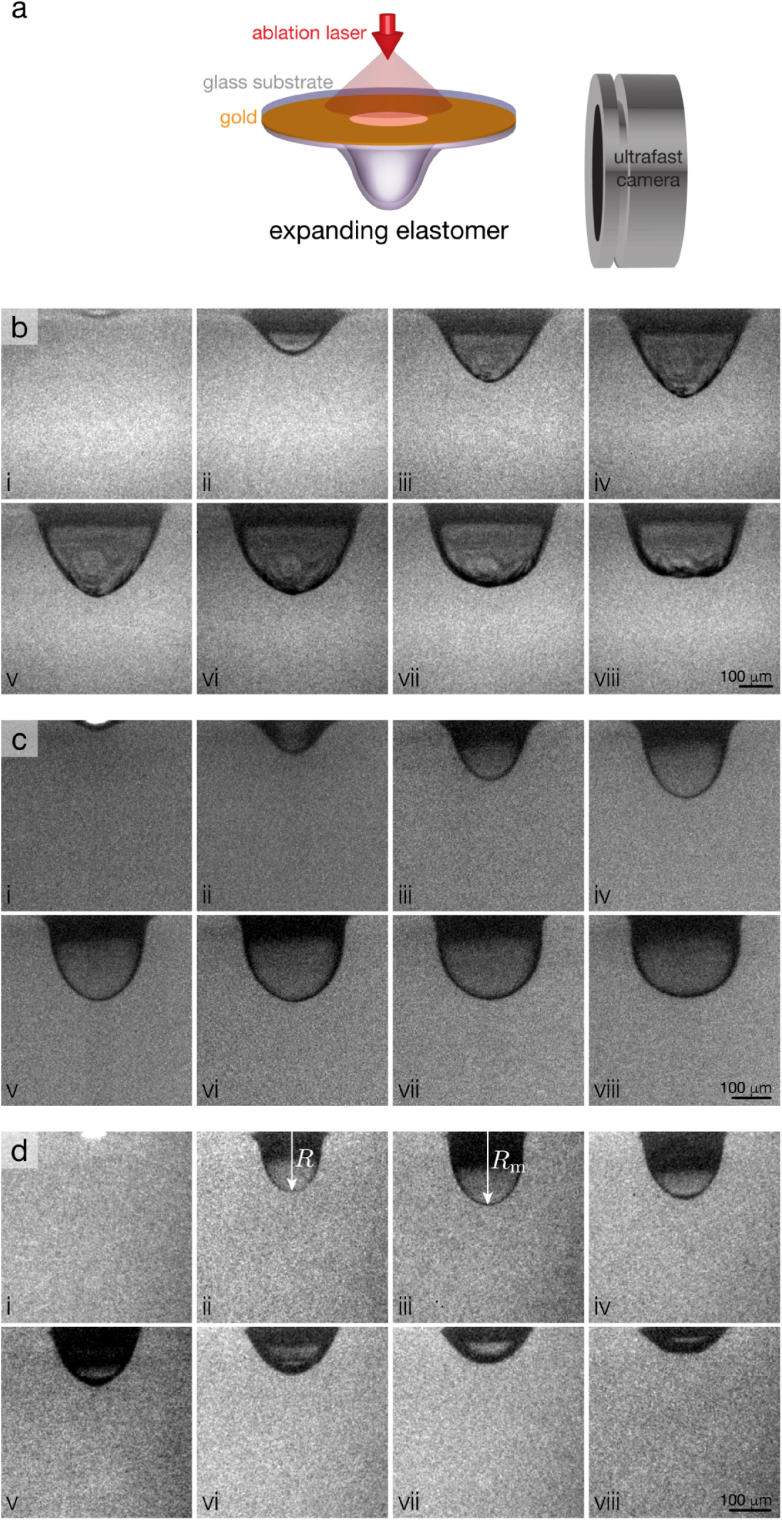
(a) Schematic of a LIME experiment. Representative LIME images of (b) *b*PDMS (*h* = 80 μm, interframe time = 500 ns), (c) *l*PDMS (*h* = 35 μm, interframe time = 200 ns), and (d) SIS (*h* = 44 μm, interframe time = 330 ns).

For this study, we chose three different elastomers: two formulations of PDMS (*l*PDMS, *b*PDMS) and an SIS block copolymer. These elastomers were selected due to their ubiquity across commercial and fundamental research applications. The two PDMS elastomers have different topologies. *l*PDMS consists of a mixture of covalent cross-links, linear entanglements, and silica filler particles. *b*PDMS consists of a linear PDMS backbone with a high density of PDMS side chains (bottlebrush), but with a small fraction of covalent cross-links connecting the PDMS bottlebrushes. *b*PDMS is not believed to contain linear entanglements.^24^ From quasi-static contact mechanical testing, the two PDMS formulations have quite different shear modulus, 412 kPa versus 36 kPa for *l*PDMS and *b*PDMS, respectively. SIS was chosen as a thermoplastic, rather than a thermoset, with a shear modulus of ≈450 kPa. Its shear modulus is comparable to that of *l*PDMS, allowing us to compare the response of a thermoset versus a thermoplastic network. Table 1 summarizes the mechanical properties of the three polymers as measured using contact mechanical testing.

**Table 1:** Young’s Storage Modulus (*E* ^†^) and Shear Storage Modulus (μ) Measured using Quasi-Static Indentation Test

Name	Material	Strain rate (s^–1^)	*E* ^†^ (kPa)	μ (kPa)	Test duration (s)
SIS	Vector 4411	0.005	1798 ± 94	454 ± 24	20
*l*PDMS	Sylgard 184 15:1 ratio	0.01	1633 ± 62	412 ± 16	20
*b*PDMS	Bottlebrush PDMS	0.007	142 ± 20	36 ± 5	51

Figure 1b,c,d shows the LIME bubble expansion process for *b*PDMS, *l*PDMS, and SIS, respectively. While these materials display different mechanical behavior, the bubble expansion processes are qualitatively similar. Taking SIS as an example (Figure 1d), the LIME experiment consists of the following steps. Initially, a plasma forms, which appears as a bright spot encapsulated by the film (panel (i)). This plasma causes the rapid expansion of the elastomer, resulting in a bubble with radius *R* (panel (ii)). The bubble continues to expand rapidly (panels iii–iv) until it reaches its maximum radius, *R*_m_, at time *t*_m_ (panel (v)). The bubble then begins to contract more slowly than it expands (panels iv–vii). The material does not return to its initial dimensions, consistent with the notion of permanent deformation of the elastomer resulting from either large strain or high strain rate. Although the bubble formation processes are qualitatively similar, *R*_m_ and *t*_m_ are elastomer and thickness-dependent.

In many of these experiments, the shape of the membrane is more oblong, which is somewhat unusual. To gain a general understanding of how polymer and geometric properties define impulsive deformation, our analysis below assumes that the bubble grows as a spherical cap. Further studies will focus on modeling the exact shape of the membrane to gain a deeper understanding of the properties that govern it. Additionally, the oblong shape of the expanding PDMS membrane is most likely attributed to the competition between adhesive interactions between the peeling edge of the membrane and the substrate, as well as the impulsive motion of the membrane. This effect will be explored systematically in a follow-up work, as it is an interesting way to study adhesion at fast deformation rates.

Figure 2a shows the radius versus time data for a 56 μm thick SIS film, which is comprised of six separate LIME experiments that were stitched together. Since the ultrafast camera is limited to collecting 12 frames for a single experiment, as shown in Figure 1b–d, we conducted multiple LIME experiments at different frame rates for a given elastomer system and film thickness and merged the data sets. This merging of separate data sets has two benefits. First, it demonstrates that each LIME experiment is very reproducible, as multiple experiments on different portions of the sample can be combined to reproduce the same dynamic response. Second, this composite data set increases the certainty that the appropriate physical model is selected to extract the relevant material properties of the elastomer.

**Figure 2:**
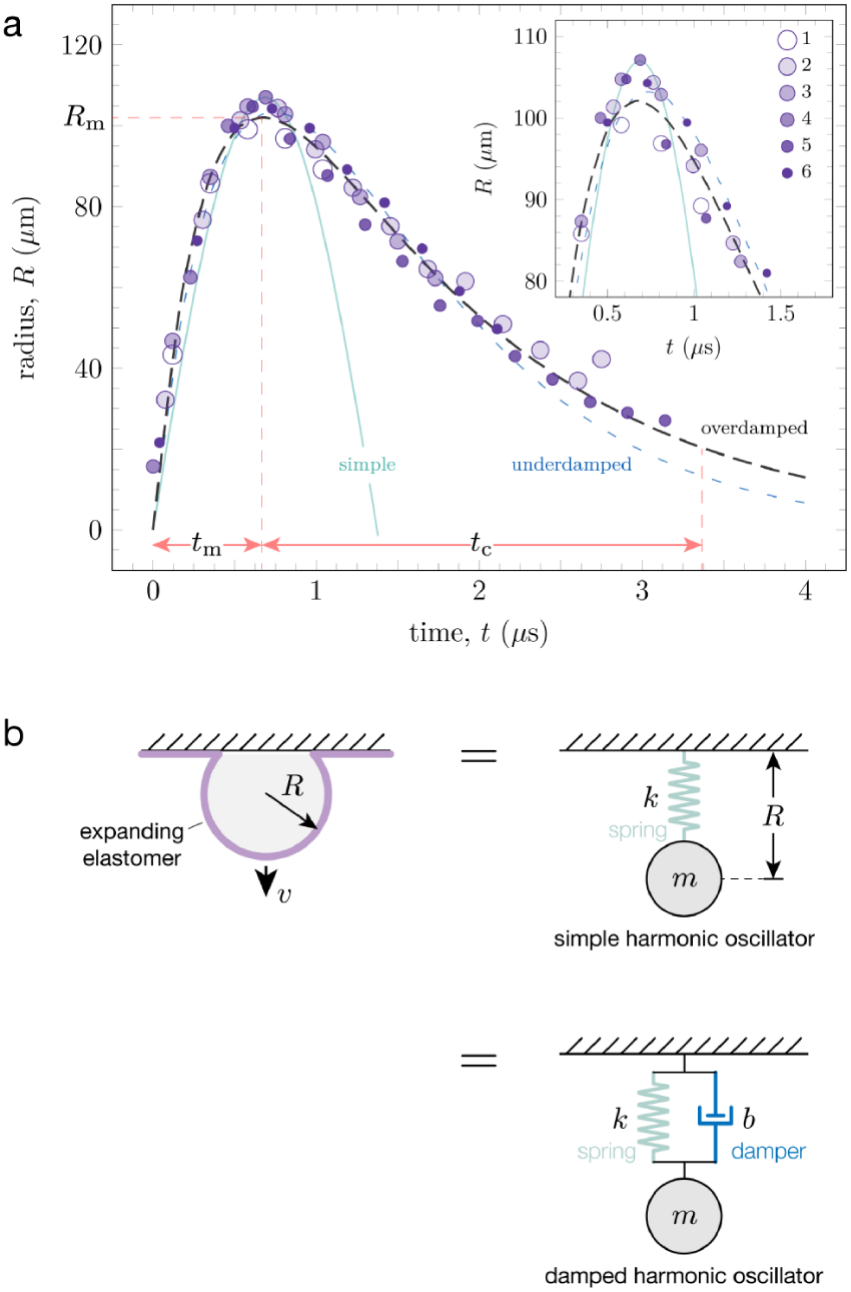
(a) Representative LIME data for SIS (*h* = 56 μm) with fits using a simple (green), underdamped (blue) and overdamped (black) harmonic oscillator model. The data set is a composite of six different LIME experiments, shown by the color gradient. Inset is a zoomed-in portion of the data with the harmonic oscillator fits at the resonant frequency when *R* = *R*_m_ and *t* = *t*_m_. (b) Schematic of the expanding elastomer and its equivalent mass-spring system depicting a simple and a damped harmonic oscillator.

Previously, dimensional analysis was used to determine the shear modulus of the elastomer by relating the rate of change of the bubble radius during the expansion process to the various materials and geometric parameters.^15^ While this approach successfully determined the shear modulus of the elastomer, it provides little insight into the underlying physics and polymer deformation mechanisms. Here, we model the LIME process as a mass-spring system to gain better insights into the material properties contributing to this impulsive deformation event (Figure 2b). Using a harmonic oscillator model enables us to depict the entire cavitation process, including expansion and retraction, thereby providing an improved understanding of how energy is stored and dissipated in these three elastomers. All relevant variables for a harmonic oscillator equation of motion are summarized in Table 2. Using this mass-spring system concept, we can equate the dynamic bubble expansion process as a harmonic oscillation process consisting of a mass element of mass *m* connected to a spring element with spring constant *k*. The simplest model is the simple harmonic oscillator, and the equation of motion is defined as

1 $$R=\frac{v_{0}}{\omega_{o}}\sin\left(\omega_{o}t\right)$$

where *R* is the bubble radius, and *t* is the elapsed time of the LIME experiment. This bubble initially expands at a rate defined by the initial velocity *v*_0_ and will oscillate indefinitely as it has no energy dissipation mechanisms.

**Table 2:** Description of Variables of a Harmonic Oscillator’s Equation of Motion

Variable	Description
*R*	Bubble radius
*t*	Time
*v*_0_	Initial velocity
ω_o_	Resonant frequency, $\omega_{o}=\sqrt{k/m}$
*k*	Spring constant
*m*	Mass
γ	Damping ratio, γ = *b*/*m*
*b*	Viscous damping coefficient
γ/ω_o_	Dissipation factor
Ω_o_	Overdamped frequency, $\Omega_{o}=\sqrt{{\left(\gamma /2\right)}^{2}-\omega_{o}^{2}}$
Ω_u_	Underdamped frequency, $\Omega_{u}=\sqrt{\omega_{o}^{2}-{\left(\gamma /2\right)}^{2}}$

However, it is clear that the bubble does not oscillate indefinitely, indicating energy is dissipated. To account for energy dissipation, a damped harmonic oscillator can be used as it contains a dissipative element, *b*, (Figure 2b) that will eventually arrest the bubble motion. Damped harmonic oscillators can be underdamped (damping energy < stored energy) or overdamped (damping energy > stored energy). A third case, a perfectly damped harmonic oscillator (damping energy = stored energy), is unlikely for nonengineered systems and is not considered in this study. The equations of motion for an underdamped harmonic oscillator and an overdamped harmonic oscillator are defined as

2 $$R=\frac{v_{0}}{\Omega_{u}}e^{(\gamma /2)t}\sin\left(\Omega_{u}t\right)$$

3 $$R=\frac{v_{0}}{\Omega_{o}}e^{(\gamma /2)t}\sinh\left(\Omega_{o}t\right)$$

The meaning of damping in a LIME experiment will be discussed later. The derivations of the three equations of motion are provided in the Supporting Information (SI).

To illustrate the importance of choosing the appropriate model, we fit the composite data set with simple (green), underdamped (blue), and overdamped (black) harmonic oscillators. As shown in Figure 2a, all three models adequately fit the expansion portion of the test. Before reaching *R* = *R*_m_, the simple harmonic oscillator model (green) predicts a slightly smaller radius than the damped model at any given time point. The second stage, when the peak bubble radius is reached (see inset), corresponds to the resonant frequency of the bubble when *R* = *R*_m_ and *t* = *t*_m_. Here, all three fits are similar, although *t*_m_ for the underdamped model (blue) is at a slightly longer time. All three models fit the bubble expansion up to *t*_m_, which suggests that energy dissipation does not significantly affect the expansion process.

The difference in the quality of the fits can be seen during bubble contraction. The simple harmonic oscillator provides an inadequate fit because it assumes no energy dissipation. As such, both the overdamped and underdamped models provide much better fits to the contraction process. The underdamped model predicts a smaller bubble radius than the data at long times. For the SIS sample shown here, the overdamped model was selected as it provided the best fit to the entire data set, based on the calculated coefficient of determination (*R* ^2^) values.

Figure 3 shows the fits for *b*PDMS, *l*PDMS, and SIS as a function of film thickness. *b*PDMS, the softest material as measured via contact mechanical testing, was fit with an underdamped model, with the exception of the 40 μm film, which will be discussed below. (Figure 3a). *l*PDMS and SIS were fit using an overdamped harmonic oscillator model (Figure 3b,c). Additionally, we remark that the radius of the bubble during contraction differs from the fit for some of the thinner films at long times. This deviation is likely due to the permanent damage experienced by the materials as they are deformed at large strains and extremely high strain rates. The damage is not so severe that the films cannot partially recover. Figure S2 shows an optical image of the surface of *l*PDMS after the LIME event, clearly showing evidence of permanent deformation on the surface of the film. The surfaces of *b*PDMS and SIS also appear similar after the LIME event. Permanent damage may complicate the exact understanding of how energy is dissipated in these elastomer systems. However, we believe that the harmonic oscillator model is appropriate as the deformation of these elastomers is still primarily elastic. Further work is underway to understand plastic deformation in LIME events, particularly for materials where plastic deformation significantly contributes to energy dissipation.

**Figure 3:**
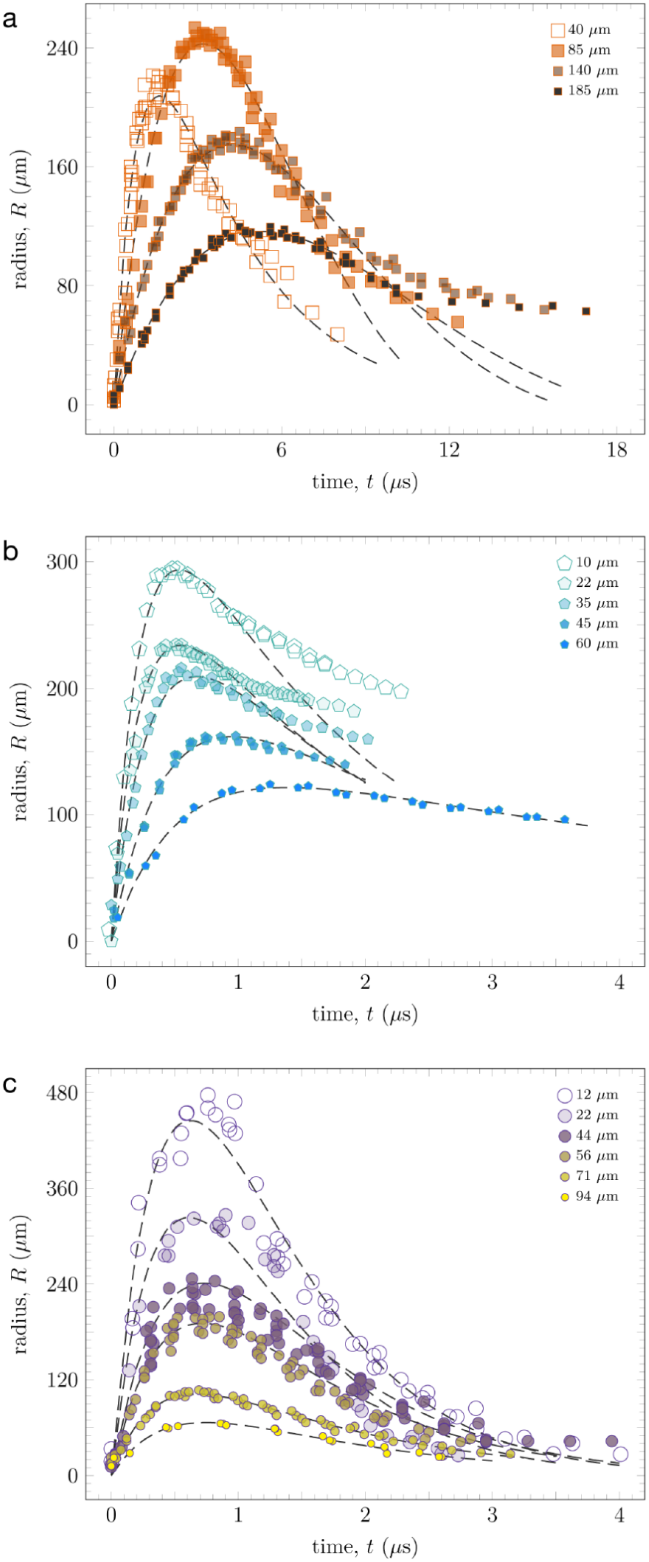
Bubble radius (*R*) versus time (*t*) data for (a) *b*PDMS, (b) *l*PDMS, and (c) SIS for the various thicknesses investigated. Also included in the plots are fits of the data using an underdamped or overdamped harmonic oscillator model with *R* ^2^ > 0.95. The radius was measured as the maximum deflection away from the substrate in each frame. Error on *t* is ±3 ns and error on *R* is ±6 μm. Laser power ≈500 mJ, except 40 μ*m b*PDMS film, ≈400 mJ.

The 40 μm film *b*PDMS is somewhat anomalous. The *R*_m_ is lower than the thicker films, and it was fitted with an overdamped harmonic oscillator model. Unlike the other films, the cavitation event for this film was induced at a reduced laser power (80%) to avoid rupture. As a result, this film does not reach as large an *R*_m_ and its resonant frequency likely shifts somewhat as a result of less energy input into the system. The volume of material involved in the bubble will also differ. However, it is unlikely the damping behavior of the film will be significantly affected, as this is intrinsic to the elastomer. As a result of lower energy input from the ablation event, this particular film was fit with an overdamped model. The exact effects of attenuating the laser power in a LIME experiment are unknown and will be examined in greater detail in further studies.

By fitting the results with the damped harmonic oscillator models, we can determine the shear modulus (μ) of the elastomer by relating the resonant frequency to the following material and geometric properties,

4 $$\mu =\frac{\omega_{o}^{2}\rho R_{m}h}{2\left(1+\nu \right)}$$

where ρ is the polymer density, *R*_m_ is the maximum bubble radius, *h* is the film thickness, and ν is the Poisson ratio. The derivation of eq 4 can be found in the SI.

Figure 4 summarizes the shear modulus of the three materials as a function of strain rate. The strain rate was estimated as ε̇ = *v*_0_/*h*, using *v*_0_ from fitting. The strain rates sampled here are ε̇ ∼ 10^5^ s^–1^ to 10^7^ s^–1^ for *b*PDMS, and ε̇ ∼ 10^6^ s^–1^ to 10^8^ s^–1^ for *l*PDMS and SIS. The shear moduli for *b*PDMS, *l*PDMS, and SIS are quite similar, despite having very different shear moduli at quasi-static testing conditions. Specifically, the shear modulus values range between μ ∼ 3 × 10^5^ Pa to 9 × 10^5^ Pa, μ ∼ 7 × 10^5^ Pa to 4 × 10^6^ Pa, and μ ∼ 2 × 10^6^ Pa to 4 × 10^6^ Pa for *b*PDMS, *l*PDMS, and SIS, respectively.

**Figure 4:**
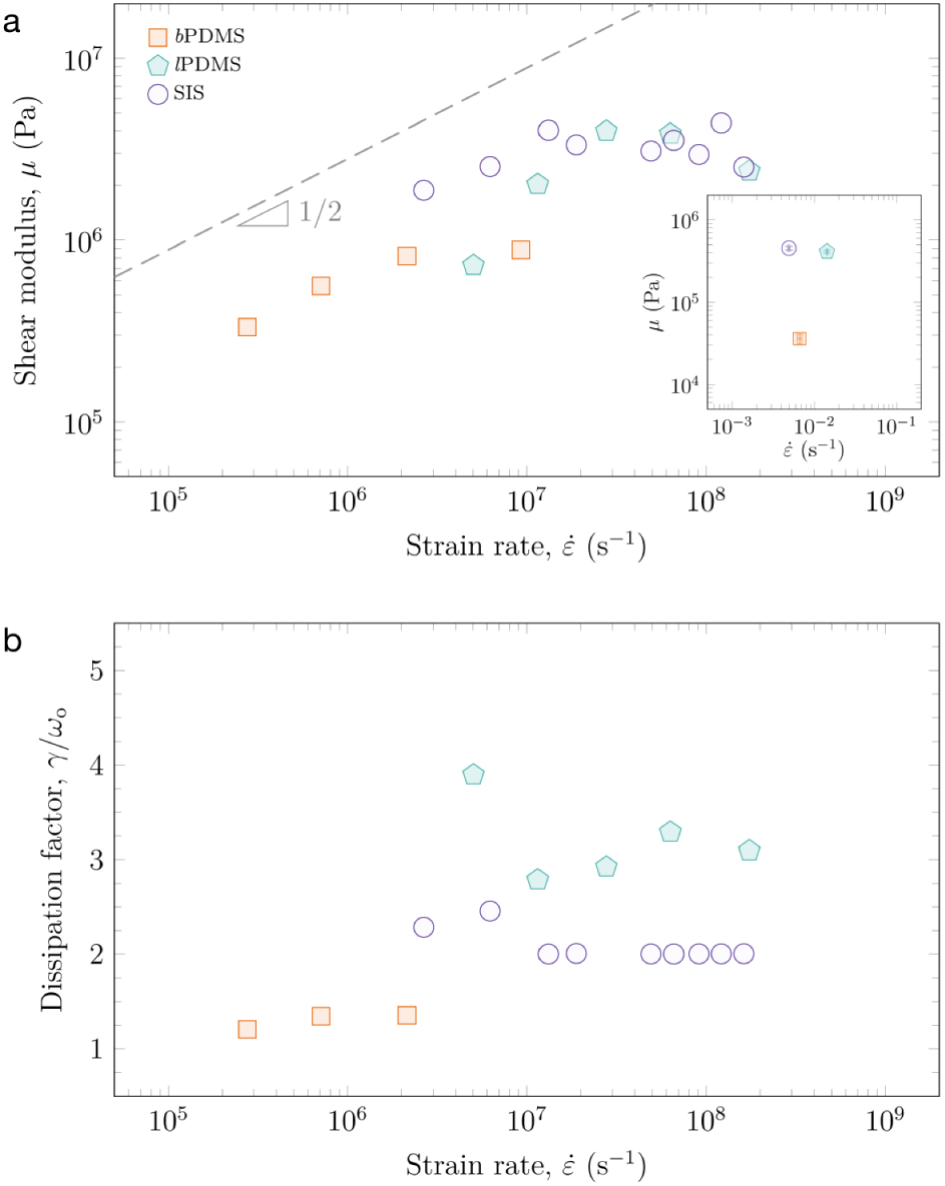
Ultrahigh strain rate mechanical properties of the three elastomer systems as measured by LIME. (a) Shear modulus (μ) vs strain rate (ε̇). The inset plot shows the shear modulus of the three elastomers measured quai-statically via contact mechanical testing. (b) Dissipation factor (γ/ω_o_) vs strain rate (ε̇). Orange squares are *b*PDMS, green hexagons are *l*PDMS, and purple circles are SIS.

The results in Figure 4a show that the shear modulus for all three materials increases with strain rate and then reaches a plateau value at a material-specific rate. The plateau in shear modulus occurs at ε̇ ∼ 2 × 10^6^ s^–1^, ε̇ ∼ 10^7^ s^–1^, and ε̇ ∼ 2 × 10^7^ s^–1^ for *b*PDMS, SIS, and *l*PDMS, respectively. The strain rate stiffening behavior also differs slightly for the materials. The *b*PDMS and SIS stiffens as μ ∼ ε̇^1/2^, whereas *l*PDMS stiffens as μ ∼ ε̇, which was reported previously.^15^ We speculate that this larger scaling exponent for *l*PDMS is likely attributed to the composite-like nature of the material. *l*PDMS is a commercial material (Sylgard 184) containing silica particles. Silica particles are added to enhance the modulus and toughen the material, so it follows that they would also affect the strain-stiffening behavior of a material.^25,26^ The increase in modulus may be due to strain rate stiffening, a well-known phenomenon in polymers.^1,3^ However, an increase in shear modulus may also be due to a temperature rise caused by adiabatic heating at these high strain rates for entropic polymer networks. Thus, we cannot discount either strain rate stiffening or temperature effects in our LIME experiments. The presence of a plateau suggests strain-rate-stiffening is the dominant phenomenon, as will be discussed below.

Besides determining the high strain rate shear mechanical properties of the elastomers, the harmonic oscillator model provides additional insights into the impulsive deformation of polymeric materials. Figure 4b shows the dissipation factor (=γ/ω_o_) for the elastomers as a function of strain rate. This parameter describes how energy released from the impulsive deformation event is stored (ω_o_, the resonant frequency) versus dissipated (γ, the damping coefficient) through the bubble expansion and contraction process.

The larger the dissipation factor, the more damping in the system. Interestingly, the dissipated energy is greater than the stored energy for all three materials as indicated by γ/ω_o_ > 1. *b*PDMS has the lowest dissipation factor, and it is nearly independent of strain rate. This could be attributed to the lower strain rates achieved; however, the dissipation factor is lower than that of the other two materials, even at the highest strain rate. The dissipation factor of *l*PDMS does not seem to have a clear trend. As the strain rate increases, it first sharply decreases, then slowly increases. Nevertheless, *l*PDMS has the highest dissipation factor across all strain rates. This is likely due, again, to its use of silica particles as filler. The dissipation factor for SIS is essentially constant for strain rates >10^7^ s^–1^, and is slightly higher before this rate. The three materials show clear differences in how they dissipate energy as a function of strain rate, which is in stark contrast to their elastic responses, which is essentially the same for *l*PDMS and SIS after ∼10^7^ s^–1^. We remark that γ/ω_o_ > 1 has important implications if energy dissipation is solely attributed to viscoelasticity. However, while dissipation is captured by the γ parameter, it is a lumped parameter, and we are unable to deconvolve this parameter into the specific dissipative mechanisms in this study. There are many possible mechanisms of energy dissipation for LIME, including the adhesion of the film at the interface, as well as the viscoelasticity of the elastomer, and other nonlinear effects of the elastomer. The harmonic oscillator model is useful for understanding the macroscopic motion of the material in these LIME events, but it lacks molecular details. These effects are important to understand energy flow and will be investigated in future studies.

## Discussion

The plateau values in the shear modulus and dissipation factor suggest a fundamental limit in the elastomer’s response to an impulsive deformation. Since this plateau begins at the same strain rate for the shear modulus and dissipation factor, we can use this critical strain rate (ε̇_o_) to calculate a monomer response time, τ_o_ = 1/ε̇_o_. We consider this time scale to be the minimum number of Kuhn monomers per chain that is mechanically active during the expansion and retraction process of the LIME event. Specifically, the segmental response time (τ_seg_) is estimated as

5 $$\tau_{\mathrm{seg}}\approx \tau_{o}N^{2}$$

where *N* is the number of monomers that comprise the load-bearing network strand.^14^ Here, the load-bearing strand does not necessarily equate to the number of monomers between cross-links or entanglements, as typically measured in quasi-static rheological or mechanical measurements. Depending on the strain rate, this load-bearing strand could span a range of segment lengths from one monomer to the length of a cross-link or entanglement strand. Here, we estimate a value for τ_seg_ for expansion, defined as the time from the start of the LIME experiment to maximum bubble expansion (=*t*_m_), and a value for τ_seg_ for retraction, defined as the time for bubble contraction (=*t*_c_). In our experiments, *t*_c_ is the time between the maximum bubble radius until the bubble reduces its size by 80% of the maximum bubble radius (=20% *R*_m_). This threshold was chosen because the bubble never fully contracts back to a flat film, as previously mentioned. We would like to emphasize that the τ_seg_ here is not directly measured by a technique such as quasielastic neutron scattering or dielectric spectroscopy, but rather it is an empirically determined average time scale that we believe governs the response of the material. τ_seg_ is known to vary depending on the technique used to measure it. We are equating this critical time scale to a fundamental time scale that defines an elastically active polymer strand to estimate *N*.

Using these time scales, we estimate the number of monomers that are elastically active during expansion and contraction (Figure 5b). A numerical example of how *N* was calculated is presented in the SI. Given the large deformation of the elastomers during expansion, all of the monomers are displaced during the LIME experiment. Here, we are quantifying the size of the load bearing strand that is active within the time scale of the LIME experiment—the elastically active polymer strand. Additionally, we find a gradual decrease in *N* with increasing strain rate for all three elastomers, which is consistent with the notion that an increase in strain rate corresponds to a reduction in the response time of the polymer (Figure S1). Focusing first on the expansion portion of the LIME experiment, we estimate that *N*  ≈2 to 3 for *b*PDMS and SIS, and *N*  ≈3 to 5 for *l*PDMS. Consistent with an impulsive deformation, the mechanical response is not associated with the collective motions of the entire polymer network. Within such short times, only a very short segment can respond to the deformation, as opposed to the entire chain.

**Figure 5:**
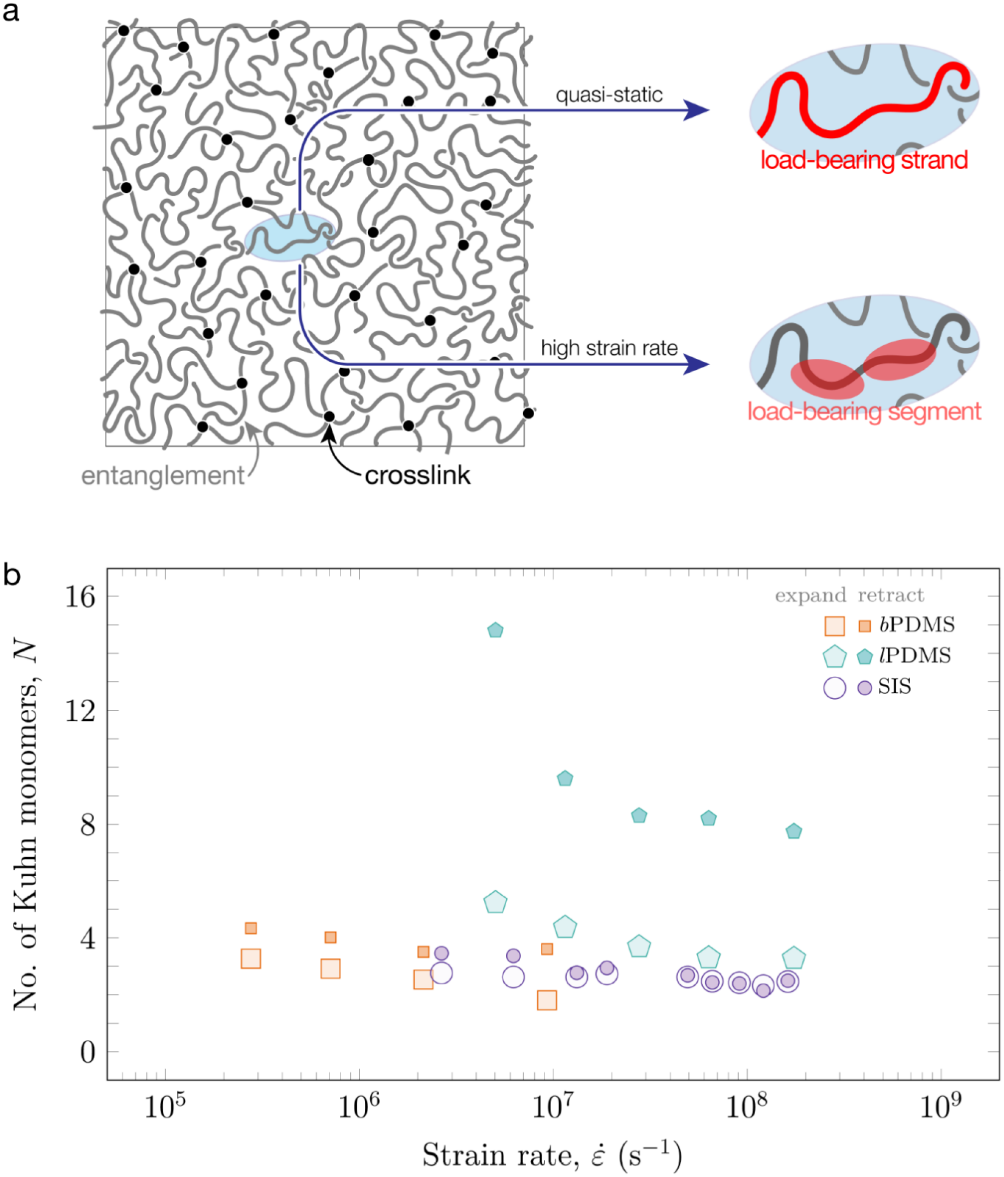
(a) Comparing the polymer chains involved in a quasi-static mechanical test (a load-bearing strand) versus a high strain rate (a load-bearing segment) test. (b) Estimation of the number of elastically active load-bearing monomers during the expansion and retraction process of the three polymers based on the LIME results.

Compared to bubble expansion, the bubble contraction is a much longer process as shown in Figure 3 and Figure S1. Importantly, our calculation of *N* for retraction represents the response time of the elastically active strand to reach 20% of *R*_m_, which is an underestimate as the maximum contraction will be even longer. With this in mind, there are a few interesting findings. First, even though the retraction times for all three elastomers are similar, we estimate that *N*  ≈3 to 4 for both *b*PDMS and SIS, whereas *N*  ≈9 to 16 for *l*PDMS. This result suggests that the filler particles present in *l*PDMS enhance polymer chain dynamics even at these short times. Additionally, the presence of fillers may also play a role in enhancing energy dissipation during an impulsive deformation event due to mechanical impedance mismatch between the rigid fillers and the soft matrix, as demonstrated recently for a similar block copolymer system.^27^ Both SIS and *l*PDMS can be approximated as such materials. For SIS, the polystyrene domains serve as effective hard fillers in the soft polyisoprene matrix. As previously stated, *l*PDMS has hard silica fillers in the soft PDMS matrix, albeit of different sizes and composition. As such, both systems have many interfaces between two materials with very different mechanical properties that can greatly impact stress wave attenuation. However, because the polystyrene “fillers” in SIS are chemically bonded in the elastomeric network, they do not appear to facilitate mobility of the polymer chain segment, even though they increase the dissipation factor noticeably as compared to *b*PDMS.

Another factor for the lower energy dissipation in *b*PDMS may be related to the relative stiffness of its Kuhn monomer. Generally, bottlebrush polymers have been shown to have large Kuhn lengths, on the order of 5 to 10 nm or more.^24^ In contrast, PDMS has a Kuhn length of 1.3 nm, and polyisoprene and polystyrene have Kuhn lengths of 0.8 and 1.8 nm, respectively.^14^ Since the entanglement molecular mass of bottlebrush polymers is >10^7^ g mol^–1^,^28^ the bottlebrush backbone is unable to dissipate energy through either chain pullout or chain disentanglement at the strain rates imposed in LIME, which here leads to lower energy dissipation compared to SIS and *l*PDMS elastomers.

We can also apply eq 5 to determine the number of Kuhn monomers involved in a quasi-static mechanical test (Table 1). For the indentation tests conducted here, the probe was in contact with *l*PDMS, *b*PDMS, and SIS for 20, 51, and 20 s, respectively. As an illustrative example, we find *N*  ≈ 6300 for *l*PDMS. This value exceeds the total number of monomers present in a single chain, suggesting the material response is attributed to the entire load-bearing strand (Figure 5a); the entanglement strand for *l*PDMS and the cross-linked strand for *b*PDMS. As discussed in the introduction, a key difference between a quasi-static and high strain rate mechanical test is the strand length that is elastically active to the imposed deformation, as discussed here and illustrated schematically in Figure 5.

Finally, our LIME results show that SIS and *l*PDMS are the most similar in the high strain rate shear modulus and dissipation factor, even when considering that *b*PDMS was measured at a slightly lower strain rate. As this study and a prior one demonstrates,^15^ the connectivity of a polymer network (thermoset versus thermoplastic) does not appear to govern the high strain rate mechanical response. Topology is typically the design parameter for controlling the mechanical properties of polymers in quasi-static mechanical loading situations. However, these results suggest that other design parameters must be considered for the high strain rate behavior of polymeric materials, such as the presence of interfaces with domains of different mechanical properties or optimizing the number of Kuhn monomers involved during an impulsive event. Further research into the exact contributions of polymer topology in the high strain rate mechanical response of soft materials is ongoing.

## Conclusions

In this work, we used LIME to study the high strain rate mechanical properties of three elastomers. By modeling the LIME event as a harmonic oscillator, we were able to quantify the shear modulus of the elastomers as a function of strain rate. Damped harmonic oscillator models were used to describe the energy dissipation of these elastomers, which we envision can be used to understand how soft materials dissipate energy during impulsive deformation events. Using scaling theory, we estimated the size of the elastically active polymer segment during the LIME event.

This work demonstrates that the mechanical properties at high-strain rates can differ significantly from those under quasi-static test conditions. The shear moduli for all three elastomers are similar at the high strain rates studied here; however, their values differ significantly when measured quasi-statically. We propose examining the segmental response time, use of fillers or other interfaces, and size and stiffness of a polymer segment as a new design parameter for materials undergoing high strain rate or impulsive deformation.

## Experimental Section

Certain equipment, instruments, software, or materials are identified in this paper in order to specify the experimental procedure adequately. Such identification is not intended to imply recommendation or endorsement of any product or service by NIST, nor is it intended to imply that the materials or equipment identified are necessarily the best available for the purpose.

### Materials

All materials were used as received. The block copolymer (BCP) was VECTOR 4111A Styrene-Isoprene-Styrene (SIS). Linear polydimethylsiloxane (PDMS, Sylgard 184) was purchased from Dow Chemicals. Reactive PDMS polymers for bottlebrush PDMS were purchased from Gelest Inc. Backbone: vinylmethylsiloxane-dimethylsiloxane copolymer trimethylsiloxy terminated, ca. 300 vinyl groups per molecule, *M*_w_ ≈ 50,000 g mol^–1^ (VDT-5035). Side chain: monohydride-terminated poly­(dimethylsiloxane), *M*_w_ ≈ 4,750 g mol^–1^ (MCR-H21). Cross-linking chain: hydride-terminated polydimethysiloxane, *M*_w_ ≈ 17,200 g mol^–1^ (DMS-H25). Glass coverslips (20 mm × 40 mm) were purchased from Corning Glass. Toluene was purchased from Avantor Sciences. Catalyst, 2% platinum in xylene (Karstedt’s catalyst) was purchased from Sigma-Aldrich.

### Polymer Preparation

Linear PDMS (*l*PDMS) was mixed in a mass ratio of 15:1 monomer:curing agent and degassed for 30 min. Bottlebrush (PDMS) (*b*PDMS) was synthesized following previously published methods.^29^ Briefly, the reactive PDMS was mixed in a molar ratio of 1:30:10 backbone:side chain:cross-linking chain with a platinum catalyst at a concentration of 5 μL g^–1^. The mixture was degassed for 30 min. SIS solution was prepared by dissolving 4111A in toluene for 48 h.

### Ablation Target Preparation

Ablation targts were prepared by sputter coating with ≈30 nm layer of gold on a glass coverslip. *l*PDMS was deposited via spin coating between 99 rad s^–1^ to 315 rad s^–1^ (for 90 s). *l*PDMS was further degassed for 30 min, then thermally cured at 70°C for 2 h. *b*PDMS films were deposited via drop casting. *b*PDMS was further degassed for 30 min, then thermally cured at 70°C for 48 h. SIS films were deposited via drop casting, then allowed to slowly dry with a 2 mL reservoir of toluene placed next to the samples under a glass dome at 21°C for 24 h.

### Contact Mechanical Test

The quasi-static modulus was quantified using a custom-built contact mechanical test instrument. The test involved displacing a spherical glass probe (2 mm diameter) toward the polymer film surface at a fixed rate (1 μm s^–1^ for SIS, 3 μm s^–1^ for *l*PDMS and *b*PDMS) until reaching a maximum compressive force (0.01 N for SIS, 0.03 N for *l*PDMS and *b*PDMS), and then immediately removing the probe at the same fixed rate. The force, displacement, and probe-film interfacial contact area was measured for the duration of the test. The effective Young’s modulus (*E* ^†^ = *E*/(1 – ν^2^), where *E* is the Young’s modulus and ν is the Poisson’s ratio was determined by fitting the force–displacement profile at the maximum interfacial contact area. The shear modulus was calculated using the relationship, μ = *E*/2­(1 + ν), where it was assumed that ν = 0.495 for all three polymers. At least four tests were conducted for each polymer. The strain rate was estimated by dividing the maximum contact radius (*a*_max_) by the displacement rate, i.e., ε̇ = δ̇/*a*_max_.

### Laser-Induced Membrane Expansion (LIME) Test

A pulsed diode-pumped solid-state IR laser (Flare NX, wavelength = 1030 nm, pulse length = 1.5 ns, Coherent Inc.) was used to ablate the gold layer of the launchpad. The laser power of approximately 500 μJ was constant across all experiments conducted. The expansion of the elastomer membranes was monitored using an ultrafast camera (SIMD12, Specialized Imaging Ltd.) with a frame rate of 10^5^ Hz to 10^6^ Hz, depending on the material and film thickness.

### Image Processing

The radius was measured as the maximum deflection away from the substrate in each frame. Error on *t* is ±3 ns and error on *R* is ±6 μm. The composite radius versus time data was fit using both an underdamped and overdamped harmonic oscillator model (see eqs 2,3). The *R* ^2^ value was used to determine which model fit better. Data points at long times were excluded to ensure the fits completely captured the resonant frequency and expansion process of the bubble.

## Supplementary Material



## Notes

Detailed derivations of the harmonic oscillator model; role of film thickness on initial membrane deformation; estimation of load-bearing strand length, and additional figures (PDF)

## References

[1] Bergström J. S., Boyce M. C. (1998). Constitutive modeling of the large strain time-dependent
behavior of elastomers. J. Mech. Phys. Solids.

[2] Wu Y.-C. M., Hu W., Sun Y., Veysset D., Kooi S. E., Nelson K. A., Swager T. M., Hsieh A. J. (2019). Unraveling the high
strain-rate dynamic stiffening in select model polyurethanes- the
role of intermolecular hydrogen bonding. Polymer.

[3] Kanyanta V., Ivankovic A. (2010). Mechanical
characterisation of polyurethane elastomer
for biomedical applications. J. Mech. Behav.
Biomed. Mater..

[4] Sheng J., Chen H., Qiang J., Li B., Wang Y. (2012). Thermal, mechanical,
and dielectric properties of a dielectric elastomer for actuator applications. J. Macromol. Sci. Part B.

[5] Kim T. K., Kim J. K., Jeong O. C. (2011). Measurement
of nonlinear mechanical
properties of PDMS elastomer. Microelectron.
Eng..

[6] Glover J. D., McLaughlin C. E., McFarland M. K., Pham J. T. (2020). Extracting uncrosslinked
material from low modulus sylgard 184 and the effect on mechanical
properties. J. Polym. Sci..

[7] Le
Bras C., Fosse C., Delbreilh L., Gervais M., Ayad M., Sounakoye A. S., Berthe L., Valadon S., Fayolle B. (2022). Transition
of elastomers from a rubber to glassy state under laser shock conditions. Soft Matter.

[8] Siviour C. R., Jordan J. L. (2016). High strain rate mechanics of polymers: A review. J. Dyn. Behav. Mater..

[9] Prathumrat P., Nikzad M., Hajizadeh E., Arablouei R., Sbarski I. (2022). Shape memory elastomers: A review
of synthesis, design,
advanced manufacturing, and emerging applications. Polym. Adv. Technol..

[10] Powell S. K., Cruz R. L. J., Ross M. T., Woodruff M. A. (2020). Past, present, and
future of soft-tissue prosthetics: Advanced polymers and advanced
manufacturing. Adv. Mater..

[11] Sun Y., Kooi S. E., Nelson K. A., Hsieh A. J., Veysset D. (2020). Impact-induced
glass-to-rubber transition of polyurea under high-velocity temperature-controlled
microparticle impact. Appl. Phys. Lett..

[12] Alarifi I. M. (2023). A comprehensive
review on advancements of elastomers for engineering applications. Adv. Ind. Eng. Polym. Res..

[13] Herzberger J., Sirrine J. M., Williams C. B., Long T. E. (2019). Polymer design for
3D printing elastomers: Recent advances in structure, properties,
and printing. Prog. Polym. Sci..

[14] Rubinstein, M. ; Colby, R. H. Polymer physics; Oxford University Press, 2008.

[15] Evans K. M., Soles C. L., Chan E. P. (2023). Studying the high-rate deformation
of soft materials via laser-induced membrane expansion. Soft Matter.

[16] Tiwari S., Kazemi-Moridani A., Zheng Y., Barney C. W., McLeod K. R., Dougan C. E., Crosby A. J., Tew G. N., Peyton S. R., Cai S., Lee J.-H. (2020). Seeded laser-induced cavitation for studying high-strain-rate
irreversible deformation of soft materials. Soft Matter.

[17] Estrada J.
B., Barajas C., Henann D. L., Johnsen E., Franck C. (2018). High strain-rate
soft material characterization via inertial cavitation. J. Mech. Phys. Solids.

[18] Palla-Papavlu A., Dinca V., Luculescu C., Shaw-Stewart J., Nagel M., Lippert T., Dinescu M. (2010). Laser induced
forward
transfer of soft materials. J. Optics.

[19] Morales M., Munoz-Martin D., Marquez A., Lauzurica S., Molpeceres C. (2018). Laser-induced
forward transfer techniques and applications. Adv. Laser Mater. Process..

[20] Delaporte P., Alloncle A.-P. (2016). Laser-induced forward transfer: A high resolution additive
manufacturing technology. Opt. Laser Technol..

[21] Chen S. H., Souna A. J., Soles C. L., Stranick S. J., Chan E. P. (2020). Using microprojectiles
to study the ballistic limit of polymer thin films. Soft Matter.

[22] Chen S. H., Souna A. J., Stranick S. J., Jhalaria M., Kumar S. K., Soles C. L., Chan E. P. (2022). Controlling toughness of polymer-grafted
nanoparticle composites for impact mitigation. Soft Matter.

[23] Veysset D., Lee J. -H., Hassani M., Kooi S. E., Thomas E. L., Nelson K. A. (2021). High-velocity micro-projectile
impact testing. Appl. Phys. Rev..

[24] Clarke B. R., Witt C. L., Ilton M., Crosby A. J., Watkins J. J., Tew G. N. (2024). Bottlebrush Networks:
A Primer for Advanced Architectures. Angew.
Chem..

[25] Nunes R., Fonseca J., Pereira M. (2000). Polymer–filler interactions
and mechanical properties of a polyurethane elastomer. Polym. Test.

[26] Liu J., Zong G., He L., Zhang Y., Liu C., Wang L. (2015). Effects of fumed and mesoporous silica nanoparticles on the properties
of sylgard 184 polydimethylsiloxane. Micromachines.

[27] Centellas P. J., Mehringer K. D., Bowman A. L., Evans K. M., Vagholkar P., Thornell T. L., Huang L., Morgan S. E., Soles C. L., Simon Y. C. (2024). Mechanochemically responsive polymer enables
shockwave visualization. Nat. Commun..

[28] Li Z., Tang M., Liang S., Zhang M., Biesold G. M., He Y., Hao S.-M., Choi W., Liu Y., Peng J. (2021). Bottlebrush
polymers: From controlled synthesis, self-assembly, properties
to applications. Prog. Polym. Sci..

[29] Cai L.-H., Kodger T. E., Guerra R. E., Pegoraro A. F., Rubinstein M., Weitz D. A. (2015). Soft polydimethylsiloxane
elastomers from architecture-driven
entanglement free design. Adv. Mater..

