# Supplementary material for: Unraveling the Relevant Length and Time Scales of Elastomers in a High Strain Rate Test
Source: Macromolecules. 2026 Jan 22;59(3):1508–16. doi: 10.1021/acs.macromol.5c02872 (PMC12895515; doi:10.1021/acs.macromol.5c02872)
Supplement: Supplementary file 1 [file ma5c02872_si_001.pdf]

Supporting Information:
Unraveling the Relevant Length and Time Scales of Elastomers in a High Strain Rate Test

Katherine M. Evans, Dustin A. Baird, Polette J. Centellas, Christopher L. Soles, and Edwin P. Chan*

National Institute of Standards and Technology, Materials Science and Engineering Division, Gaithersburg, 20899, USA

E-mail: edwin.chan@nist.gov

1 Derivation of Harmonic Oscillator Equations of Motion

1.1 Simple Harmonic Oscillator

Simple harmonic oscillators have no dissipation. The equation of motion for a simple harmonic oscillator for a spring-mass system of mass m and spring constant k is defined as,

$$\begin{aligned} F &= ma \\ &= m\frac{\mathrm{d}^2R}{\mathrm{d}t^2} = -kR \\ \implies m\frac{\mathrm{d}^2R}{\mathrm{d}t^2} &+ kR = 0 \\ \implies \frac{\mathrm{d}^2R}{\mathrm{d}t^2} &+ \omega_0^2 R = 0 \end{aligned} \tag{1}$$

where a is acceleration, t is time, and $\omega_0 = \sqrt{k/m}$ is the resonant frequency.

The general solution to this second-order ordinary differential equation is

$$\begin{aligned} R(t) &= c_1 e^{i\omega_0 t} + c_2 e^{-i\omega_0 t} \\ &= c_1\big(\cos\omega_0 t + i\sin\omega_0 t\big) + c_2(\cos\omega_0 t + i\sin\omega_0 t\big) \\ &= (c_1 + c_2)\cos\omega_0 t + i(c_1 - c_2)\sin\omega_0 t \\ &= C_1\cos\omega_0 t + C_2\sin\omega_0 t \\ &= \sqrt{C_1^2 + C_2^2}\left(\frac{C_1}{\sqrt{C_1^2 + C_2^2}}\cos\omega_0 t + \frac{C_2}{\sqrt{C_1^2 + C_2^2}}\sin\omega_0 t\right) \\ &= A\big(\cos\omega_0 t\cos\phi + \sin\omega_0 t\sin\phi\big) \\ &= A\cos(\omega_0 t - \phi) \\ &= A\sin(\omega_0 t) \end{aligned} \tag{2}$$

Here, we arrived at this solution by assuming that the solution is: $R(t) \sim e^{\lambda t}$ (Note: **Euler's identity:** $e^{0+i\omega_0 t} = \cos\omega_0 t + i\sin\omega_0 t$, **Law of Cosines:** $\cos(\alpha - \beta) = \cos\alpha\cos\beta + \sin\alpha\sin\beta$), where C_1 and C_2 are constants that depend on the initial conditions (velocity and position at $t = 0$).

Assuming the following initial conditions, $R(0) = 0$ and $v(0) = v_0$, we obtain,

$$\begin{aligned} R(t) &= \frac{v_0}{\omega_0}\sin(\omega_0 t) \\ &= R_{\text{max}}\sin(\omega_0 t) \end{aligned} \tag{3}$$

Since there are no dissipative processes, this system will continue oscillating indefinitely at ω_0.

1.2 Damped Harmonic Oscillator

A more realistic mechanical model to depict the deformation of an elastomer for a Laser-induced membrane expansion (LIME) experiment is the damped harmonic oscillator, which includes a damping term that dissipates energy due to a friction force ($F_f = -bv$).

$$\begin{aligned} m\frac{\mathrm{d}^2R}{\mathrm{d}t^2} + b\frac{\mathrm{d}R}{\mathrm{d}t} + kR = 0 \\ \implies \frac{\mathrm{d}^2R}{\mathrm{d}t^2} + \gamma\frac{\mathrm{d}R}{dt} + \omega_0^2 R = 0 \end{aligned} \tag{4}$$

where b is the viscous damping coefficient, and $\gamma = b/m$ is the damping ratio. The general solution to this second-order ordinary differential equation is,

$$R(t) = e^{-(\gamma/2)t}\left(C_1 e^{\left(\sqrt{(\gamma/2)^2-\omega_0^2}\right)t} + C_2 e^{-\left(\sqrt{(\gamma/2)^2-\omega_0^2}\right)t}\right) \tag{5}$$

Note that this friction force does not clearly indicate whether the energy dissipation is due to adhesion between the elastomer-substrate interface, the dissipation due to air drag, or viscoelastic effects due to deformation of the elastomer.

1.2.1 Over-damped System

An over-damped oscillator, $(\gamma/2)^2 - \omega_0^2 > 0$, is one where the system returns to equilibrium without oscillating. The damping ratio determines how long the system takes to reach equilibrium, which can be

longer than the critcally-damped system. Eq. 5 becomes,

$$R(t) = e^{-(\gamma/2)t}\Big(C_1 e^{\Omega_o t} + C_2 e^{-\Omega_o t}\Big) \tag{6}$$

where $\Omega_o = \sqrt{(\gamma/2)^2 - \omega_0^2}$. If we assume that $R(0) = 0$ and $v(0) = v_0$, we obtain,

$$\begin{aligned} R(t) &= \frac{v_0}{2\Omega_o} e^{-(\gamma/2)t}\Big(e^{\Omega_o t} - e^{-\Omega_o t}\Big) \\ &= \frac{v_0}{\Omega_o} e^{-(\gamma/2)t} \sinh\big(\Omega_o t\big) \end{aligned} \tag{7}$$

1.2.2 Critically-damped System

A critically-damped system, $(\gamma/2)^2 - \omega_0^2 = 0$ and $\implies \omega_0 = \gamma/2$, is one where the system returns to equilibrium as quickly as possible without oscillating. The general solution is,

$$R(t) = e^{-(\gamma/2)t}\Big(C_1 + C_2 t\Big) \tag{8}$$

If we assume that $R(0) = 0$ and $v(0) = v_0$, we obtain,

$$R(t) = \big(v_0 t\big) e^{-(\gamma/2)t} \tag{9}$$

If we assume that $R(0) = R_0$ and $v(0) = v_0$, we obtain,

$$R(t) = \Big(R_0 + \big(v_0 - \omega_0 R_0\big)t\Big) e^{-(\gamma/2)t} \tag{10}$$

Note that since $\gamma/2 = \omega_0$ for a critically damped system, we have already substituted this into Eq. 10. A critically damped system is extremely unlikely to occur in this LIME experiment where the damping is intrinsic to the material rather than engineered.

1.2.3 Under-damped System

An underdamped system, $(\gamma/2)^2 - \omega_0^2 < 0$, is one where the system gradually returns to equilibrium via a combination of oscillation and dissipation. If we consider that $\Omega_\mathrm{u} = \sqrt{\omega_0^2 - (\gamma/2)^2}$, Eq. 5 becomes,

$$R(t) = e^{-(\gamma/2)t}\left(c_1 e^{i\Omega_\mathrm{u} t} + c_2 e^{-i\Omega_\mathrm{u} t}\right) \tag{11}$$

The general solution to this system is,

$$R(t) = e^{-(\gamma/2)t}\Big(C_1 \cos\big(\Omega_\mathrm{u} t\big) + C_2 \sin\big(\Omega_\mathrm{u} t\big)\Big) \tag{12}$$

If we assume that $R(0) = 0$ and $v(0) = v_0$, we obtain,

$$R(t) = \frac{v_0}{\Omega_\mathrm{u}} e^{-(\gamma/2)t} \sin\big(\Omega_\mathrm{u} t\big) \tag{13}$$

Notice that Eq. 13 has a similar functional form as Eq. 1 but with the addition of a decay exponential function.

1.3 Estimating Shear Modulus

The resonant frequency in a spring-mass system can be used to estimate the shear modulus by relating the spring constant k and mass m to the properties of the material.

$$\omega_0 = \sqrt{\frac{k}{m}} \tag{14}$$

This expression can be rewritten in terms of the parameters that can be obtained from a LIME experiment. Specifically, the spring constant can be rewritten as,

$$k = \frac{F}{x} = \frac{EA}{R_\mathrm{m}} \tag{15}$$

where E is the Young's modulus, A is the surface area of the deformed, and R_m is the maximum radius of the bubble. The mass of the film cannot be measured directly; thus, we approximate it to the dimensions of the deformed film,

$$m = \rho A h \tag{16}$$

where ρ is the density of the polymer and h is the film thickness. Combining Eqs. 15 and 16, we obtain,

$$\omega_0 \approx \sqrt{\frac{2\mu(1+\nu)}{\rho R_{\mathrm{m}} h}} \tag{17}$$

where we relate the Young's modulus (E) to the shear modulus (μ) assuming the polymer is an isotropic elastic solid ($E = 2\mu(1+\nu)$) with Poisson ratio ν.

1.3.1 Parabolic Membrane Deformation

Alternatively, we can estimate the mass of the deforming polymer by approximating the membrane shape with a parabolic cross-sectional profile. The velocity profile of such an expanding membrane is,

$$v(r) = v_{r=0}\left(1 - \left(\frac{r}{R}\right)^2\right), \quad 0 \leq r \leq R \tag{18}$$

With this assumption, the kinetic energy of the deformed membrane (E_{k}) is obtained by integrating over the membrane surface:

$$\begin{aligned} E_{\mathrm{k}} &= \frac{1}{2}\int_0^{2\pi}\int_0^R \rho h \big(v(r)\big)^2 \, r \, dr \, d\theta \\ &= \frac{1}{2}\left(\frac{\pi}{3}\rho R^2 h\right) v_{r=0}^2 \end{aligned} \tag{19}$$

yielding an effective mass, $m_{\mathrm{eff}} = \frac{\pi}{3}\rho R^2 h$. The surface area of a parabola, assuming the base is twice its height, is $A_{\mathrm{eff}} = 2R^2/3$. Comparing this effective mass with Eq. 16, we find that $m = (4/\pi) m_{\mathrm{eff}}$. Therefore, $m \approx m_{\mathrm{eff}}$.

1.4 Quality Factor

The quality factor (Q) quantifies the amount of damping of an oscillator. For a harmonic oscillator, it is defined as the ratio of the energy stored to the energy dissipated over one cycle,

$$Q = 2\pi \frac{E}{\Delta E} \tag{20}$$

where E is the the total energy of the system, $E = E_p + E_k$, whch is defined as the sum of the potential (E_p) and kinetic (E_k) energy,

$$\begin{aligned} E_p &= \frac{1}{2}kR^2 = \frac{1}{2}m\omega_0^2 R^2 \\ &= \frac{1}{2}m\omega_0^2 \left(\frac{v_0}{\Omega_\mathrm{u}} e^{-(\gamma/2)t} \sin\left(\Omega_\mathrm{u} t\right) \right)^2 \end{aligned} \tag{21}$$

$$\begin{aligned} E_k &= \frac{1}{2}mv^2 \\ &= \frac{1}{2}mv_0^2 e^{-\gamma t} \left(\cos\left(\Omega_\mathrm{u} t\right) - \frac{\gamma}{2\Omega_\mathrm{u}} \sin\left(\Omega_\mathrm{u} t\right) \right)^2 \end{aligned} \tag{22}$$

The total energy is then defined as,

$$\begin{aligned} E &= E_p + E_k \\ &= \frac{1}{2}m\omega_0^2 \left(\frac{v_0}{\Omega_\mathrm{u}} e^{-(\gamma/2)t} \sin\left(\Omega_\mathrm{u} t\right) \right)^2 \\ &= \frac{1}{2}mv_0^2 e^{-\gamma t} \left(\frac{\omega_0}{\Omega_\mathrm{u}} \right)^2 \left[\sin^2\left(\Omega_\mathrm{u} t\right) + \left(\frac{\Omega_\mathrm{u}}{\omega_0} \right)^2 \cos^2\left(\Omega_\mathrm{u} t\right) - \frac{\gamma\Omega_\mathrm{u}}{\omega_0^2} \sin\left(\Omega_\mathrm{u} t\right) \cos\left(\Omega_\mathrm{u} t\right) + \frac{\gamma\Omega_\mathrm{u}}{\omega_0^2} \sin^2\left(\Omega_\mathrm{u} t\right) \right] \end{aligned} \tag{23}$$

For a system that is weakly damping ($\gamma \ll \Omega_\mathrm{u}, \implies \Omega_\mathrm{u} \approx \omega_0$), the Eq. 23 simplifies to,

$$E \approx \frac{1}{2}mv_0^2 e^{-\gamma t} \tag{24}$$

Substituting Eq. 24 into Eq. 20, and evaluating the expression for one period (T), we obtain,

$$
\begin{aligned}
Q &= 2\pi \frac{E(t)}{E(t) - E(t + \mathrm{T})} \\
&= \frac{2\pi}{1 - E(t + \mathrm{T})/E(t)} \\
&= \frac{2\pi}{1 - e^{-\gamma(t+\mathrm{T})}/e^{-\gamma t}} \\
&= \frac{2\pi}{1 - e^{-\gamma \mathrm{T}}}
\end{aligned}
\tag{25}
$$

Here, we can define $\mathrm{T} = 2\pi/\gamma$ and recognize the Taylor expansion, $e^x = 1 + x + \frac{x^2}{2!} + \frac{x^3}{3!} + ...$ Then, Eq. 25 becomes,

$$
\begin{aligned}
Q &= \frac{2\pi}{1 - e^{-2\pi\gamma/\Omega_\mathrm{u}}} \\
&\approx \frac{2\pi}{1 - (1 - 2\pi\gamma/\Omega_\mathrm{u} + ...)} \\
&\approx \frac{\Omega_\mathrm{u}}{\gamma} \\
&\approx \frac{\omega_0}{\gamma}
\end{aligned}
\tag{26}
$$

Table S1 summarizes the γ/ω_o values extracted from the LIME experiments.

Table S1: Extracted dissipation factor values (γ/ω_o) as a function of strain rate ($\dot{\varepsilon}$) from LIME testing of the three polymer systems.

Name	Material	$\dot{\varepsilon}$ (s^{-1})	γ/ω_o
SIS	Vector 4411	4.93×10^7	2.00
		2.65×10^6	2.28
		6.23×10^6	2.46
		6.60×10^7	2.00
		9.11×10^7	2.00
		1.21×10^8	2.00
		1.62×10^8	2.00
		1.89×10^7	2.01
		1.32×10^7	2.00
*l*PDMS	Sylgard 184 15:1 ratio	1.74×10^8	3.10
		6.32×10^7	3.29
		2.77×10^7	2.92
		1.15×10^7	2.79
		5.03×10^6	3.90
*b*PDMS	Bottlebrush PDMS	2.14×10^6	1.36
		7.10×10^5	1.35
		2.76×10^5	1.21

1.4.1 Viscoelasticity

We can also use the quality factor to describe the dissipation of viscoelastic systems. In general, it is related to the real ($\mu'(\omega)$) and imaginary ($\mu''(\omega)$) component of the shear modulus,

$$Q = \frac{\mu'(\omega)}{\mu''(\omega)} \tag{27}$$

Also, recall that $\tan\delta = \mu''/\mu'$. Thus, Q for a viscoelastic system describes how "elastic" the system is. A Q-factor < 1, or $\gamma/\omega_0 > 1$, implies that viscous dissipation is present. However, for LIME experiments, we cannot solely attribute a large value of γ/ω_0 to viscoelasticity of the polymer as other dissipative processes such as adhesion between the polymer and the substrate contributes to the energy dissipation.

2 Role of Film Thickness on the Initial Membrane Deformation

We can quantitatively describe the relationship between the initial membrane velocity and membrane thickness by approximating the initial membrane expansion process as similar to the pressurization of a thin spherical shell. The stress experienced by this thin spherical shell is defined by the hoop stress σ_{h}, which is related to the thickness (h) and radius (a) as,

$$\sigma_{\mathrm{h}} = \frac{\Delta P}{2}\frac{a}{h} \tag{28}$$

where $\Delta P = P_{\mathrm{i}} - P_{\mathrm{o}}$ is the pressure inside the shell. Since LIME is an impulsive deformation event that involves shock wave propagation, we can relate the hoop stress in terms of the shock impact stress,

$$\sigma_{\mathrm{i}} = \frac{1}{2}\rho C_{\mathrm{S}} V \tag{29}$$

where ρ and C_S are the density and acoustic wave speed of the material, respectively. Setting Eq. 28 equal to Eq. 29, we obtain a relationship between velocity and membrane thickness.

$$V_\mathrm{o} = \frac{\Delta P}{\rho C_\mathrm{S}} \frac{a_\mathrm{o}}{h} \tag{30}$$

Note that we have ignored the effects of adhesion, which will increase the critical pressure required to expand the membrane $\sigma_\mathrm{h} = \Delta P \frac{a_\mathrm{o}}{h} - \sigma_\mathrm{adh}$.

Alternatively, we can relate the initial membrane velocity in terms of ablation energy E_ab,

$$V_\mathrm{o} = \left(\frac{E_\mathrm{ab}}{\rho h a_\mathrm{o}^2} \right)^{1/2} \tag{31}$$

3 Estimating Load-Bearing Strand Length

We estimate the size of the load-bearing strand from the results shown in Figure 4 and Figure S1. The results in Figure 4 show that both the shear modulus and dissipation factor reach a plateau at a critical strain rate ($\dot{\varepsilon}_\mathrm{o}$) for a given material. We assume that this critical point reflects the fastest rate or shortest time (τ_o) the shortest polymer strand, *i.e.,* the Kuhn monomer, can respond to the impulsive deformation. Thus, we assume that $\tau_\mathrm{o} \approx 1/\dot{\varepsilon}_\mathrm{o}$. From Figure 4, we find that the plateau values in shear modulus occurs at $\dot{\varepsilon}_\mathrm{o} \sim 2 \times 10^6$ s^{-1}, $\dot{\varepsilon}_\mathrm{o} \sim 10^7$ s^{-1}, and $\dot{\varepsilon}_\mathrm{o} \sim 2 \times 10^7$ s^{-1} for *b*PDMS, SIS, and *l*PDMS, respectively.

The segmental response time (τ_seg) is related to this shortest time as, $\tau_\mathrm{seg} \approx \tau_\mathrm{o} N^2$, where N is the number of Kuhn monomers that comprise the load-bearing network strand.[1] Due to the inertial nature of LIME test, the strain rate of the test is not a fixed quantity like a quasi-static mechanical test. Instead, we estimate a value for τ_seg for expansion, defined as the time from the start of the LIME experiment to maximum bubble expansion ($t_\mathrm{m} = \tau_\mathrm{seg}$), and a value for τ_seg for retraction, defined as the time for bubble contraction ($t_\mathrm{c} = \tau_\mathrm{seg}$).

As a numerical example, we will evaluate the *b*PDMS system. From Figure 4a, we estimate that $\tau_\mathrm{o} \approx 1/2 \times 10^6$ s for this polymer. From Figure S1a, $t_\mathrm{m} = 1.62$ μs translates to $\tau_\mathrm{seg} = 1.62 \times 10^{-6}$ s, thus yielding $N \approx \sqrt{\tau_\mathrm{seg}/\tau_\mathrm{o}} \approx \sqrt{1.62 \times 10^{-6} \cdot 2 \times 10^6} = 1.8$ Kuhn monomers. Similarly, from Figure S1b,

$t_c = 6.44$ μs yields $N \approx \sqrt{\tau_{seg}/\tau_o} \approx \sqrt{6.44 \times 10^{-6} \cdot 2 \times 10^6} = 3.6$ Kuhn monomers.

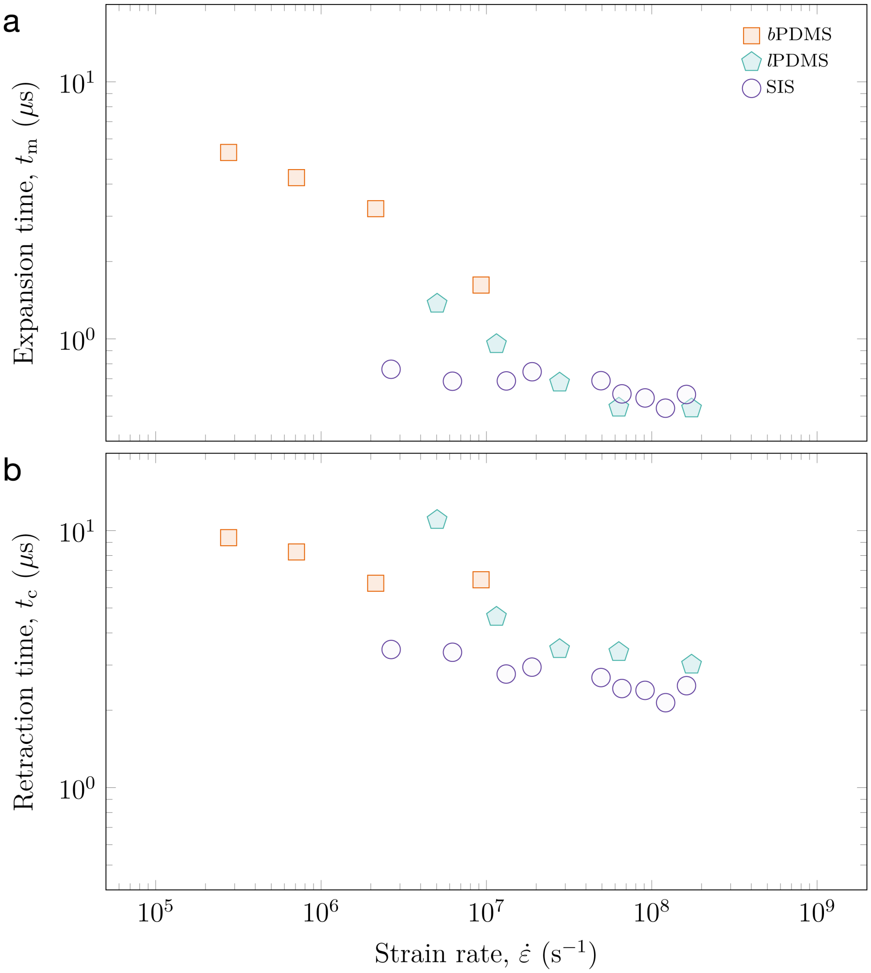

Figure S1: The time required for a LIME bubble to a) expand to R_m (t_m), and b) retract to 20 % R_m (t_c) as a function of strain rate for the three elastomers.

4 Additional Figure

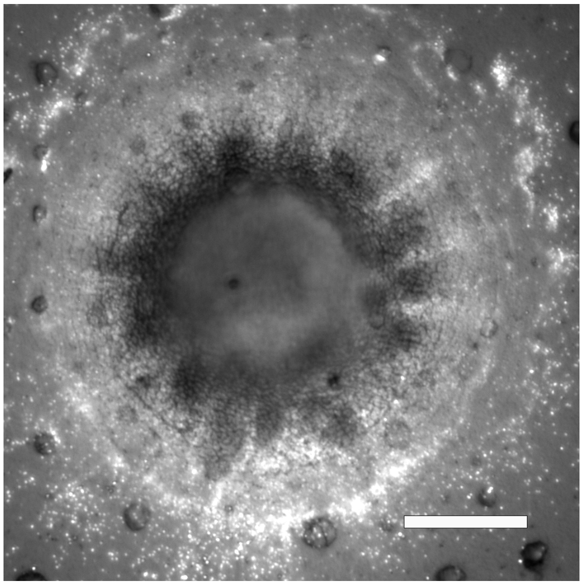

Figure S2: Optical image of a 22 μm PDMS film after the LIME event. Scale bar=25 μm.

References

(1) Rubinstein, M.; Colby, R. H. *Polymer physics*; Oxford University Press, 2008.
